# Animal models of major depression: drawbacks and challenges

**DOI:** 10.1007/s00702-019-02084-y

**Published:** 2019-10-04

**Authors:** Barbara Planchez, Alexandre Surget, Catherine Belzung

**Affiliations:** 1 UMR 1253, iBrain, Université de Tours, Inserm, Tours, France; 2 UMR 1253, iBrain, UFR Sciences et Techniques, Parc Grandmont, 37200 Tours, France

**Keywords:** Depression, Antidepressant, Treatment-resistant depression, Animal models, Validity

## Abstract

Major depression is a leading contributor to the global burden of disease. This situation is mainly related to the chronicity and/or recurrence of the disorder, and to poor response to antidepressant therapy. Progress in this area requires valid animal models. Current models are based either on manipulating the environment to which rodents are exposed (during the developmental period or adulthood) or biological underpinnings (i.e. gene deletion or overexpression of candidate genes, targeted lesions of brain areas, optogenetic control of specific neuronal populations, etc.). These manipulations can alter specific behavioural and biological outcomes that can be related to different symptomatic and pathophysiological dimensions of major depression. However, animal models of major depression display substantial shortcomings that contribute to the lack of innovative pharmacological approaches in recent decades and which hamper our capabilities to investigate treatment-resistant depression. Here, we discuss the validity of these models, review putative models of treatment-resistant depression, major depression subtypes and recurrent depression. Furthermore, we identify future challenges regarding new paradigms such as those proposing dimensional rather than categorical approaches to depression.

## Introduction

Major depressive disorder (MDD) is one of the main contributors to the global burden of disease (Ledford 2014). Indeed, it is the leading cause of disability as measured by years lived with disability (YLDs) and is currently ranked second in disability adjusted life years (DALY) calculated for all ages.

However, it is not a homogenous pathology and according to symptomatology and associated biological changes, different subtypes of MDD have been described, including melancholic and atypical depression. MDD is a recurrent condition, in which each episode increases the probability of a further episode (Association 2000; Solomon et al. 2000), a condition termed as recurrent depression. Some researchers have proposed a dimensional framework (Cuthbert and Insel 2013), in which the different psychiatric disorders can be described according to different transnosographic domains (the Research Domain Criteria or RDoC). Within this framework, depression is mostly related to two of the RDoC domains: the loss construct and various reward constructs within the domains of Negative Valence Systems and Positive Valence Systems, respectively (Woody and Gibb 2015).

In general, MDD is initially treated with chronic antidepressants (ADs), namely drugs whose main effect is to increase monoaminergic neurotransmission either by blocking the serotoninergic and/or the noradrenergic transporter (selective serotonin or noradrenaline reuptake inhibitors, or tricyclic ADs) or by blocking their degradation by the monoamine oxidase inhibitors. However, after a 2–3-month treatment period, remission rates are low, ranging from 20 to 40% in naturalistic studies (Cuffel et al. 2003; Trivedi et al. 2006; Rush et al. 2006) to 40–60% in randomised trials (Cipriani et al. 2007). Further on, some patients do not show remission after treatments with several ADs: resistance to pharmacotherapy is conventionally referred to as treatment-resistant depression (TRD). However, even TRD does not remain without treatment option as recently, the Food and Drug Administration (FDA) has approved non-monoaminergic drugs such as esketamine (an antagonist of the NMDA receptor) and brexanolone (a positive allosteric modulator of the GABA-A receptors) for postpartum depression.

In recent decades, progress has been made regarding the understanding of the psychological constructs underlying MDD, the description of brain circuits showing abnormal information processing in MDD as well as the characterization of the cellular and molecular alterations associated with this disorder (Willner et al. 2013; Belzung et al. 2015; Willner and Belzung 2015). This progress has been made possible in part through the use of rodent models, which have allowed the roles of candidate neural circuits, neurophysiological systems and molecular targets to be investigated, like, for example, abnormalities in the stress axis or increased neuro-inflammation. However, fewer advances have been achieved regarding innovative treatments, as hardly any novel pharmacological classes have been introduced since the 1990s. This may be related to a poor understanding of the criteria of validity of animal models. For example, an animal model of MDD is considered relevant and valid only if it shows responses to currently used AD therapies, which (1) implies the risk of merely finding molecules with action mechanisms akin to conventional ADs, and (2) hampers the development of animal models of TRD, as a validity criterion of TRD should be that rather than responding to conventional AD treatments, it privileges responses to treatments of TRD such as neurostimulation. The validity of an animal model is usually assessed through several criteria. A classical view proposes three criteria: predictive validity, face validity and construct validity (Willner 1984; Belzung and Lemoine 2011). (1) Predictive validity refers to specific and selective responsiveness to treatments. This means that if a drug is effective in MDD patients under certain conditions, it should act in the same way in the animal model. For example, if an AD is acting only after chronic but not acute treatment in the clinical population, it should also be ineffective after a single injection in the rodent model, but reveal its effectiveness only after chronic administration. The model should also reproduce the treatment resistance: a certain percentage of subjects of the animal population should, thus, exhibit TRD, and this is rarely assessed. Further on, animals exhibiting TRD should respond to esketamine or to neurostimulation, as in the clinical condition. (2) Face validity corresponds to the phenomenological similarities between the animal model and the human condition: comparing the symptomatology between the human condition and alterations of specific behavioural endpoints resulting from experimental manipulations related to the animal model of MDD should enable this aspect to be assessed. For example, many patients with MDD exhibit anhedonia (Buckner et al. 2008); therefore, it is essential to include anhedonia in the behavioural tests carried out on rodents representing an animal model of depression. However, here again the situation is complex, as the symptoms of patients with MDD are very heterogeneous, with a high degree of within-disorder variability, so that patients with the same diagnosis may share few or even no symptoms (Krueger and Bezdjian 2009; Olbert et al. 2014). The model should also recapitulate the biological alterations found in the clinical condition, which are expressed, for example, by changes in some peripheral biomarkers such as levels of cortisol or corticosterone in humans or rodents, respectively. However, these criteria may be difficult to meet, because, as for behavioural symptoms, differences between patients may be great not only in terms of biological systems that are affected but also in the direction of the changes observed in a particular system (e.g. divergent endocrine abnormalities in melancholic and atypical depression: clinical and pathophysiological implications: Gold and Chrousos 2002). (3) Finally, construct validity means that the model has a sound theoretical rationale such as equivalence of the causative or triggering factors of the disease (i.e. vulnerability genes and environmental factors), similarity of psychological constructs associated with MDD, including alterations in self-referential schemas and cognitive bias (Belzung et al. 2015), and of the underlying neurobiological mechanisms including alterations of the processing in prefrontal areas and the cingulate cortex, amygdala, lateral habenula and hippocampus.

In recent years, there has been a lack of meaningful advances in the search for novel therapeutic strategies against MDD; thus, the aim of this review is to: (1) describe current rodent models of MDD and also behavioural endpoints used to assess their effects; (2) discuss the validity of the current rodent models of MDD; and (3) discuss putative models of TRD, subtypes of MDD and recurrent depression, and identify future challenges. These different aspects are epitomised by results from the rodent model literature, using indistinctly findings from rat and mouse model. It is beyond the purpose of this review to underline precisely the differences between rats and mice for each model. However, it is important to remind that there are notable physiological, anatomical, biochemical and pharmacological differences between rats and mice and, hence, a same model or molecule may work differently in both species (Ellenbroek and Youn 2016).

## Behavioural endpoints and animal models of MDD

### Behavioural endpoints

**Fig. 1:**
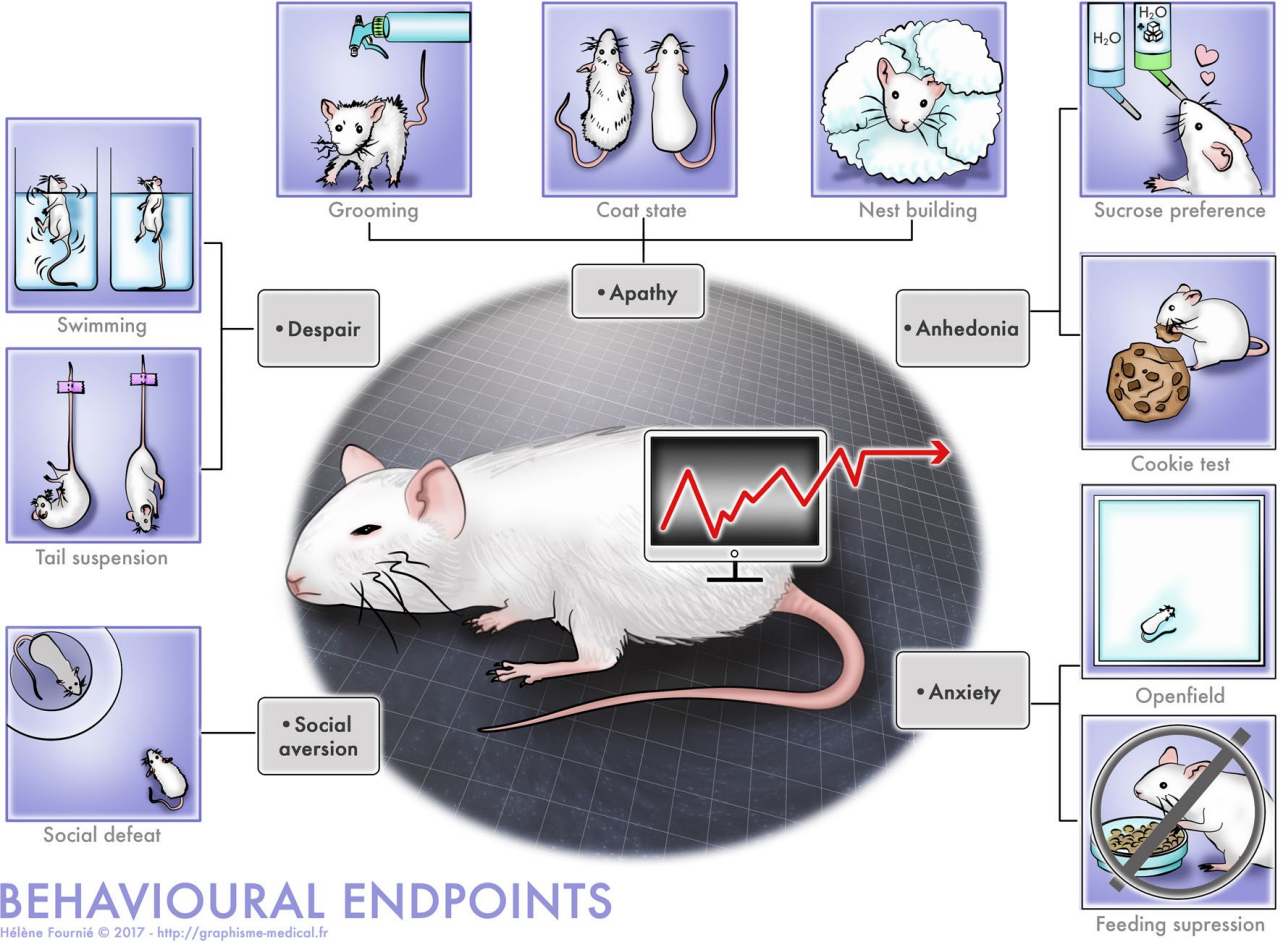
Behavioural endpoints measuring aspect of the anxio-depressive phenotype in rodents

Symptoms of MDD include core symptoms (anhedonia and depressed mood) as well as additional symptoms (sleep disturbance, changes in weight/appetite and psychomotor alteration) and other associated conditions such as anxiety and social withdrawal (Hasler et al. 2004), that can be easily assessed in animals: therefore, various tests have been designed to measure these different aspects (Fig. 1). While many behavioural situations exist to assess anhedonia (see below), the situation is more complex regarding sadness. In behavioural terms, depressed mood (or sadness) is generally expressed by social withdrawal, slow gait, and disengagement: although not strictly equivalent, disengagement can correspond to despair behaviour and social withdrawal, while slow gait can correspond to apathy and psychomotor retardation (Levy and Dubois 2006). We have, however, to mention that some symptoms observed in humans, such as feelings of worthlessness or excessive guilt or recurrent thoughts of death, suicidal ideation or a suicide attempt cannot be observed in animals such as rodents, and may, therefore, be absent from the behavioural endpoints that are highlighted. For this reason, the behavioural endpoints only partially reproduce the clinical condition.

#### Anhedonia

In general, anhedonia is assessed using palatable solutions or food, although more complex measures of anhedonia do exist, for example modification of the threshold of intracranial stimulation (Cryan et al. 2003). Two examples are the sucrose preference test in which animals have the choice between tap water or water containing sucrose/saccharose, and the cookie test in which animals are offered a chocolate cookie instead of regular pellets. Normal animals show a preference for the palatable food/solution, which is significantly reduced in anhedonic animals (Klein 1974). Sexual behaviours can also be used to assess anhedonia, but this is quite complicated: indeed, as female rodents are not proceptive at any time, to measure sexual behaviour in male rodents, it is necessary to use females that have previously undergone surgical ablation of their ovaries and hormone treatment. Finally, intra-cranial self-stimulation can also be used, but this requires surgery to implant stimulation electrodes within hedonic hotspots within the brain.

#### Despair

Common tests for assessing despair-like behaviours include forced swim and tail suspension tests. Both tests consist in placing a rodent in an uncomfortable situation (a water tank or a position in which the animal is suspended by its tail) from which escape is impossible. At the beginning, animals exhibit active behaviours (swimming or struggling). However, because the situation is inescapable, at some point, the animals start displaying bouts of immobility, which then incrementally rise. Early on, it became evident that these protocols were easily implemented and demonstrated that single AD injections or subchronic treatments could reduce immobility in both tests (Porsolt et al. 1977; Steru et al. 1985), and this lead to the rapid spread of their use in laboratories focusing on depression. They have become a gold standard for the early screening of novel molecules with putative AD-like effects. At the same time, immobility started to be commonly qualified as “despair” and considered to reflect depression-like states.

However, such utilizations rely mainly on reverse inferences, with the risk of interpretation bias or fallacy. Indeed, it is not because conventional ADs may promote immobility that either a decrease or an increase in immobility induced by a novel drug, a gene variant or a model can be selectively explained through either depression-like or AD-like effects, respectively. Many alternative explanations can also underlie such effects and are frequently lacking in the literature. Moreover, immobility could actually be interpreted as an adaptive behaviour, as it enables the animal to cope with the situation without spending energy in a useless manner. In fact, some models of depression have been associated with greater struggling in inescapable situations (Tronche et al. 1999; Lu et al. 2008; Surget et al. 2016), suggesting that a greater caution should be taken when interpreting results from these tests and encourage researchers to use more appropriate controls to strengthen the validity of their claims.

Thus, screening potential novel therapeutic strategies or assessing depression-like states of animal models uniquely with such tests is far from being sufficient and requires other symptomatic dimensions of the depressive syndrome to be included.

#### Hopelessness

A method to observe hopelessness is the learned helplessness paradigm (Seligman 1972). In this procedure, rodents first receive several electric shocks on their feet in a closed chamber. Then, the subjects are placed in another chamber with a grid floor and receive a mild shock with the possibility to escape. Rodents that have not been previously exposed to the unescapable shock are commonly able to escape quickly from the shock, whereas animals previously exposed to the learned helplessness paradigm frequently fail to acquire shock avoidance (Maier and Seligman 2016). Two characteristics are important for the development of hopelessness: the unpredictability and the uncontrollability of the stressors (here the shocks). While frequently confounded, these two concepts can be separated as situations exist in which a subject has few control over a situation that is perfectly predictable (for example a stressor that is applied routinely and from which the subject cannot escape) as well as situations in which a subject has a control over events that he/her cannot predict (he/her can stop the occurrence of the stressor when it occurs, for example, with a lever in a skinner box). It has been shown that controllability or predictability is sufficient to mitigate the development of helplessness (Burger and Arkin 1980).

#### Apathy

Apathy has been defined as a deficit in goal-directed behaviour (Levy and Dubois 2006). In rodents, five different measurements have been proposed to assess it, including impaired nest building, disturbed self-grooming, reduced maternal care, reduced social interest and reduced interest for novel objects (Cathomas et al. 2015). Two aspects have been measured in the context of animal models of MDD: nest building and grooming, either using coat state deterioration or the splash test. It is necessary to measure spontaneous activity in parallel, as a decline in activity can have nonspecific effects on spontaneous behaviour. In brief, the measure of the coat state deterioration consists in assessing the state of the fur on seven different parts of the body of a mouse: when the animal is stressed, it might show coat state deterioration (ruffled or dirty coat). The splash test consists in splashing a sucrose solution over the coat of the animal and measuring the grooming behaviour that has been induced.

#### Anxiety

Many devices have been designed to assess anxiety-related behaviours in rodents (Gould et al. 2009). However, in the context of MDD, two situations are mainly used: forced confrontation of a rodent with a new environment (e.g. an open field, an elevated plus maze, a light/dark box or a free exploration test) and placing the animal in a situation with a motivational conflict (e.g. novelty suppression of feeding, NSF, conflict between the drive to eat a food pellet and the avoidance of open space).

#### Abnormalities in eating behaviour

Most depressive patients exhibit changes in food consumption and weight, either with loss or gain of appetite (Hasler et al. 2004). Many measures can be performed to analyse such abnormalities in rodents; the animals can either be weighed regularly to observe a potential loss/gain, or the relative lack/increase of weight gain in comparison to non-stressed animals can be measured. In addition, food intake can be assessed by providing a palatable diet directly in the rodents’ home cage: a precise portion is given and the intake is measured (Ulrich-Lai et al. 2015; de Souza et al. 2018). On the other hand, some tests initially developed to assess endpoints such as anxiety or anhedonia can provide information concerning eating behaviour. The NSF test consists in measuring the latency to eat in a novel environment, reflecting the level of anxiety; but in this case, once the mice start to eat, they are taken back to their home cage with the pellets and their feed intake is measured for five more minutes. Similarly, in the cookie test initially designed to assess anhedonia, consumption of the cookie can be measured, thus evaluating appetite (Dadomo et al. 2011). While anhedonia translates into a decrease of palatable food consumption, a change in appetite translates into a change in food intake, whatever its palatability.

#### Sleep disturbance

As in depressive patients, rodents can exhibit disruption of sleep patterns following chronic stress, which can be evaluated during sleep periods either by EEG (slow wave rhythm) or rapid eye movement (REM) sleep. On the other hand, the evaluation of diurnal ambulatory behaviours can reflect the general level of activity which can be affected by changes in sleep rhythm (Vanderheyden et al. 2015; Sickmann et al. 2018; Cui et al. 2018; Ding et al. 2018).

#### Psychomotor agitation or retardation

The level of general activity can be modified in depression, reflected by agitation or retardation in human patients. Some animal models of depression also exhibit psychomotor abnormalities (Vollmayr and Henn 2003); the evaluation of home cage locomotor activity can give an idea of activity levels without any interference due to anxiety or exploration of a novel environment (Dadomo et al. 2011). In addition, nest building activity can also be used to assess daily-living behaviours. Nevertheless, locomotor activity can be recorded in a novel environment such as an open field, an elevated plus maze or a light–dark box to measure reactivity. Moreover, the forced swim test has also been used to assess retardation reflected by exaggerated immobility (Overstreet and Wegener 2013).

#### Irritability

Although irritable mood is only considered as a core symptom in children and adolescents, studies have found that irritability is highly prevalent in adult depression (Kovess-Masfety et al. 2013; Judd et al. 2013). Additionally, anger and irritability are one of the key symptoms in the diagnoses of some depression subtypes such as melancholic depression and premenstrual dysphoric disorder in women (American Psychiatric Association 2013; Hantsoo and Epperson 2015). Irritability in human and rodents is partially associated with dysfunctional and aberrant response to threat (Leibenluft 2017). To assess this paradigm in animal models of depression, the resident–intruder test can be used to measure the level of aggressiveness/irritability when confronted to a potential threat, in this case a conspecific placed in the home cage (Ho et al. 2001; Mineur et al. 2003; Schneider and Popik 2007a). The burying marble test has also been proposed to evaluate irritability, although it is usually characterised as a model of anxiety and compulsive behaviours in rodents (Njung’e and Handley 1991; Angoa-Pérez et al. 2013); it could reflect irritability if we consider the exaggerated burying behaviour as an aberrant threat response toward harmless objects (Schneider and Popik 2009). In this paradigm, the animal is placed in a cage with sawdust containing nine marbles: once the animal is removed, the buried marbles are counted and the more marbles are buried, the more the animal exhibits abnormal behaviours. Moreover, this behaviour can be decreased by acute administration of SSRIs (Schneider and Popik 2007b, 2009).

#### Cognitive impairment

Cognitive dysfunction is a core pathological feature in MDD, although most of the current treatments focus on mood dysregulation. Memory deficits and learning difficulties are often observed in depressive patients as well as deficits in attention and flexibility (McDermott and Ebmeier 2009; Zuckerman et al. 2018). These symptoms are not specific to a depressive state and many behavioural tests can assess their occurrence in animal. Different tests, protocols and settings have been used in the literature to evaluate precisely a myriad of aspects of memory, learning and cognitive impairments. It is beyond the scope of this review to detail these tests, we will just present below some examples of the most common tests to investigate such aspects in animal models of depression.

Non-social and social memory can be assessed with the object recognition test (ORT) (Leger et al. 2013) and the social recognition test (SRT) (Winslow 2003; Lemaire 2004): both tasks are based on the natural drive of rodents to explore more thoroughly new stimuli. In the ORT, the time spent to explore a new object will be measured and in the SRT, interaction time with the newcomers is evaluated. Among the standard paradigms to test for spatial memory and learning, the Morris water maze (MWM) is often used in animal models of depression (Aisa et al. 2007; Do Couto et al. 2012; Darcet et al. 2014); in the MWM, rodents are tasked with finding a platform hidden underneath the water and to learn its location, which assesses hippocampal-dependent memory because it involves learning the spatial location of the platform (Vorhees and Williams 2006). One of the most common tests for working memory is the Y maze, which relies on the natural tendency of rodents to explore new environments, and thus the three arms of the apparatus (Lalonde 2002; Coburn-Litvak et al. 2003): the percentage of spontaneous alternations is based on the frequency of complete alternations between the three arms. Associative learning can be assessed with fear conditioning: in this case, a neutral stimulus, such as a sound or a specific context, will be associated with an aversive stimulus (e.g. an electrical footshock); following the conditioning, the sole occurrence of the neutral stimulus will elicit fear responses. However, if the animal is exposed several times to the neutral stimulus without the aversive one, a new association will be learnt, i.e. the sound or the context will no longer predict the footshock: this dissociation is referred as fear extinction.

Depressive patients also elicit attentional and executive function deficits (Zuckerman et al. 2018). To evaluate flexibility and attention, reversal learning is often employed, which is based on the discrimination between two stimuli or spatial locations: one stimulus is associated with a reward while the other is not. Once the discrimination is well established, the rules are reversed, which means that the reward-associated stimulus is changed. This paradigm can easily be transposed to other species: originally developed in primates, reversal learning has been adapted to rats and mice (Izquierdo et al. 2017). Otherwise, the MWM can measure spatial flexibility if the hidden platform is moved to a new location. Additionally, other devices exist such as touch-sensitive screens bringing a wider variety of visual stimuli: this particular task relies on stimulus-reward learning with the measure of the frequency of nosepoking the reward-associated stimulus on the screen. Once the discrimination phase is complete, the reversal learning can be assessed by modifying the stimuli associated with the reward. Interestingly, studies on rats and mice highlighted similarities in the neuronal basis of reversal learning and attention with humans: the lesion or the inhibition of the orbitofrontal cortex in rats and mice induces deficits in reversal learning (Birrell et al. 2000; Bissonette and Powell 2012; Graybeal et al. 2014; Izquierdo et al. 2017).

### Animal models

**Fig. 2:**
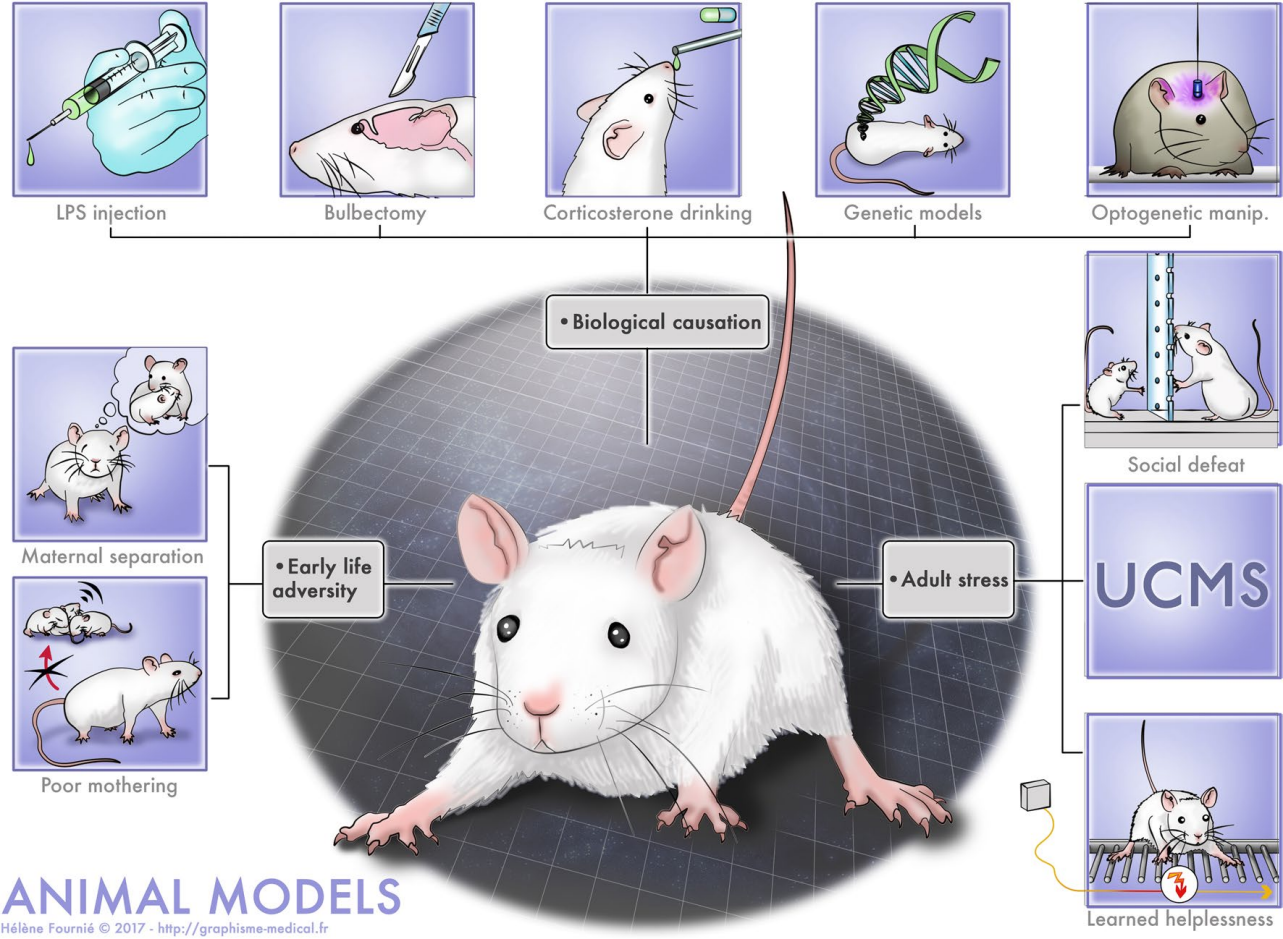
Animal models of depression. The models are mimicking different causes: early life adversity, biological causation, stress at adulthood

The fact that initial episodes of MDD are precipitated by adversity such as stress (Kendler et al. 1999; Kessler 2002) is well documented. Therefore, many animal models of MDD are based on the application of stressors, either during the developmental period or during adulthood. However, some models also recapitulate other possible aetiologies of MDD, and directly target the underlying biological substrates of MDD, such as alterations in the brain circuitry, in the stress axis and, in the immune system. These models are depicted in Fig. 2.

### Models based on application of stressors

These models consist either in applying stressors during the developmental period or adulthood, reproducing early life adversity and adverse life events, respectively.

#### Early life adversity

Rodents such as rats or mice are born at a very immature state of development, and thus, they depend strongly on maternal care. Early maternal separation is, therefore, a stressful event that may shape the behavioural and the biological phenotype of the offspring once adult (for a review, see Andersen 2015). The most popular separation procedure consists of a 3-h daily separation from the second to the 12th day postpartum. The behaviour and biological endpoints are then assessed in the animals once adult. At a behavioural level, this experimental manipulation induces deficits in learning and memory (mostly when applied to mice from the BALB/c strain), depressive-like behaviours (in BALB/c and in C57BL/6 mice) and anxiety-like behaviours (in both strains) (Vetulani 2013; Tractenberg et al. 2016). Similar findings have been demonstrated when this procedure is applied to rats (Garner et al. 2007; Aisa et al. 2007; Marais et al. 2008; Oomen et al. 2010; Bai et al. 2012). However, it is noteworthy that few studies using this protocol have found anhedonic behaviours in rodents that have been subjected to maternal separation, indicating that this model does not recapitulate all aspects of MDD (see Willner and Belzung 2015). At the biological level, these manipulations induce changes (mainly decreases) in neurotrophins such as brain neurotrophic factor: BDNF, an increase in corticosterone levels and a decrease in corticotrophin releasing factor (CRF) signalling pathways (see Holmes et al. 2005; Tractenberg et al. 2016), increased activity of some brain areas involved in stress-processing, for example, increased c-fos in the amygdala (Benner et al. 2014), increased FosB in the paraventricular nucleus (PVN) and in the amygdala (Tsuda and Ogawa 2012) and changes in neurotransmission such as modification in noradrenergic receptors (Coccurello et al. 2014). Similar findings have been obtained in rodents that undergo spontaneous deficits in maternal care, such as the ones that can be found in some strains of mice (Calatayud and Belzung 2001; Calatayud et al. 2004), in the offsprings of rats showing poor licking/grooming (Turecki and Meaney 2016) or in offsprings of rodents whose mothers were subjected to limited bedding and nesting material (Walker et al. 2017). Altogether, these studies support and extend findings from human research indicating that maternal neglect or a history of childhood abuse has devastating long-lasting consequences with, in particular, increased MDD linked to structural, epigenetic and transcriptomic changes (Lutz et al. 2017). Interestingly, few studies have revealed any effect of ADs after early stress adversity, indicating that these paradigms might instead model TRD (Willner and Belzung 2015). However, one limit is that most protocols use newborn pups whose development stage corresponds more to a pre-natal human stage, thus suggesting that the use of juvenile rodents would be more relevant.

#### Stress applied during adulthood

Different procedures have been proposed which consist in applying uncontrollable and/or unpredictable stressors to rodents. These stressors are usually repeated over several hours (learned helplessness), over days (social defeat) or over weeks (unpredictable chronic mild stress: UCMS) and they can be either mild (as, for example, in UCMS) or more intense (social defeat or learned helplessness).

##### Learned helplessness

Learned helplessness was first described by Martin Seligman at the University of Pennsylvania in 1967 (Overmier and Seligman 1967; Seligman and Maier 1967). He had observed that when dogs received inescapable foot shocks, they later fail to learn to avoid escapable foot shocks, a behaviour that he subsequently termed as learned helplessness (Seligman 1972). Furthermore, they exhibit anhedonia and despair behaviour (for reviews, see Anisman and Merali 2001). Later, these findings were extended to other species, including rodents. However, it is to be noted that not all rodents display helplessness: some are resilient, while other are vulnerable (Kim et al. 2016). The neurobiological underpinnings have been reviewed in Maier and Seligman (Maier and Seligman 2016). In sum, inescapable shock induces a strong activation of the dorsal raphe nucleus 5-HT neurons, leading to (a) an acute release of 5-HT in structures to which it projects such as the amygdala, dorsal periaqueductal grey and nucleus accumbens, (b) a long-lasting desensitisation of 5-HT1A auto-receptors in the dorsal Raphe. This activation of the dorsal Raphe does not occur after escapable shocks, as this feature is detected by the ventromedial prefrontal cortex that inhibits the dorsal Raphe. Other changes have also been detected, including changes in neurotrophins (Song et al. 2006; Shirayama et al. 2015) and increased corticosterone levels (Song et al. 2006). Interestingly, using functional neuroimaging, similar neurobiological alterations have been found in both healthy human subjects placed in uncontrollable situations and in patients with MDD, which highlights the validity of this model. However, the predictive validity of this model is low, as AD treatment elicits rapid “therapeutic-like” effects (after subchronic treatment), and as some compounds not eliciting remission in patients have shown positive effects in the animal model (reviewed in Ramaker and Dulawa 2017).

##### Social defeats

In this paradigm, the test mouse is placed in the home cage of an aggressive resident mouse 10 min daily. The test animal is, thus, attacked by the resident animal and in some cases injured (Avitsur et al. 2001; Merlot et al. 2003; Becker et al. 2008). Furthermore, the test mouse is forced to live the rest of the day in visual, olfactive and auditive but not physical contact with its aggressor. This sequence is repeated for 10 days, with a novel opponent each day. After 10 days, the animals display a behaviour characterised by social withdrawal and anhedonia. Social defeat produces some neurobiological changes relevant to MDD such as dysregulation of the prefrontal cortex (PFC), which induces increased amygdala activity (Hultman et al. 2016), release of pro-inflammatory cytokines (Reader et al. 2015), hypercortisolemia (Han et al. 2017) and changes in neurotrophins (Berton et al. 2006; Tsankova et al. 2006). Even if this procedure is more relative to male rodents, as it is more difficult to carry out the social defeat procedure in females due to their lower aggressiveness, recent studies have shown that vicarious experiences of social defeat by female mice can elicit depressive-like behaviours. Indeed, these females have decreased sucrose preference, increased immobility in the tail suspension test (TST), and also physiological abnormalities such as increased corticosterone and decreased body weight (Iñiguez et al. 2018). Interestingly, in the social defeat model, two populations of mice can be identified: one showing social avoidance, termed as susceptible mice, and the other not showing this profile, termed as resilient mice. The neural underpinning of resilience involves a wide range of brain areas including the ventral tegmental area (VTA), the prefrontal cortex (PFC), the nucleus accumbens (NAc), the central nucleus of the amygdala, the bed nucleus of the stria terminalis (BNST), the locus coeruleus and the hippocampus (Russo et al. 2012; Russo and Nestler 2013; Isingrini et al. 2016). Finally, the chronic social defeat model is sensitive to chronic SSRIs (Tsankova et al. 2006; Vialou et al. 2010) and acute ketamine treatments (Donahue et al. 2014). More details can be found in Hammels et al. (2015).

##### Chronic social instability

As mentioned, the social defeat model is not efficient to elicit depressive-like behaviours in females except for some paradigms in which the females can witness the aggression (Iñiguez et al. 2018). Indeed, females have lower defensive scores when confronted to their pairs. However, while women are more vulnerable to psychosocial stress and are more prompt to develop depressive-like behaviours (Kessler 2003; Albert 2015; Kuehner 2017), a serious dearth in female-based animal models of depression persists (Beery and Zucker 2011). This caveat has been explored in some studies to develop a relevant social depression model for females: interestingly, chronic social instability in females can elicit depressive-like symptoms in rats (Haller et al. 1999; Herzog et al. 2009; Goñi-Balentziaga et al. 2018) and mice (Tamashiro et al. 2005; Saavedra-Rodríguez and Feig 2013; Goñi-Balentziaga et al. 2018). This paradigm consists of several weeks of social instability such as an alternation of isolation/crowding phases lasting from 1 to 48 h depending on protocols. Such procedures can lead to a decrease in appetite, changes in the circadian cycle, an elevation of adrenal and corticosterone levels and a decrease of sucrose preference which reflects anhedonia (Herzog et al. 2009; Goñi-Balentziaga et al. 2018). Additionally, this paradigm has also been tested on male rats, inducing an alteration of hippocampal neurogenesis and deficits in spatial learning (McCormick et al. 2012). Moreover, a recent study showed that chronic instability during adolescence can induce anxiety-like behaviours in male rats. Nevertheless, chronic social instability shows some contradictory results among studies, probably due to discrepancies between protocols: future research should be careful to design reproducible protocols that can be compared between studies (for review see Goñi-Balentziaga et al. 2018).

##### Predator stress

Similar to the confrontation with an aggressive peer in the social defeat model, a depressive-like phenotype can be induced by exposure to a predator. Indeed, several studies showed that this type of psychological stress can lead to depressive-like behaviours reflected in decreased sucrose preference, anxiety-like behaviours in open field and social interaction tasks and finally alterations in adult neurogenesis (Burgado et al. 2014; Wu et al. 2019). These effects were counteracted by chronic treatments with fluoxetine (Wu et al. 2019). Nevertheless, this model mimics a trauma, and therefore, it should instead be considered as a model of post-traumatic stress disorder, a disorder which also responds to chronic antidepressants. This indicates that response to antidepressants cannot be considered as sufficient to determine that a given model is a model of depression, as it is non-specific.

##### Unpredictable chronic mild stress (UCMS)

**Fig. 3:**
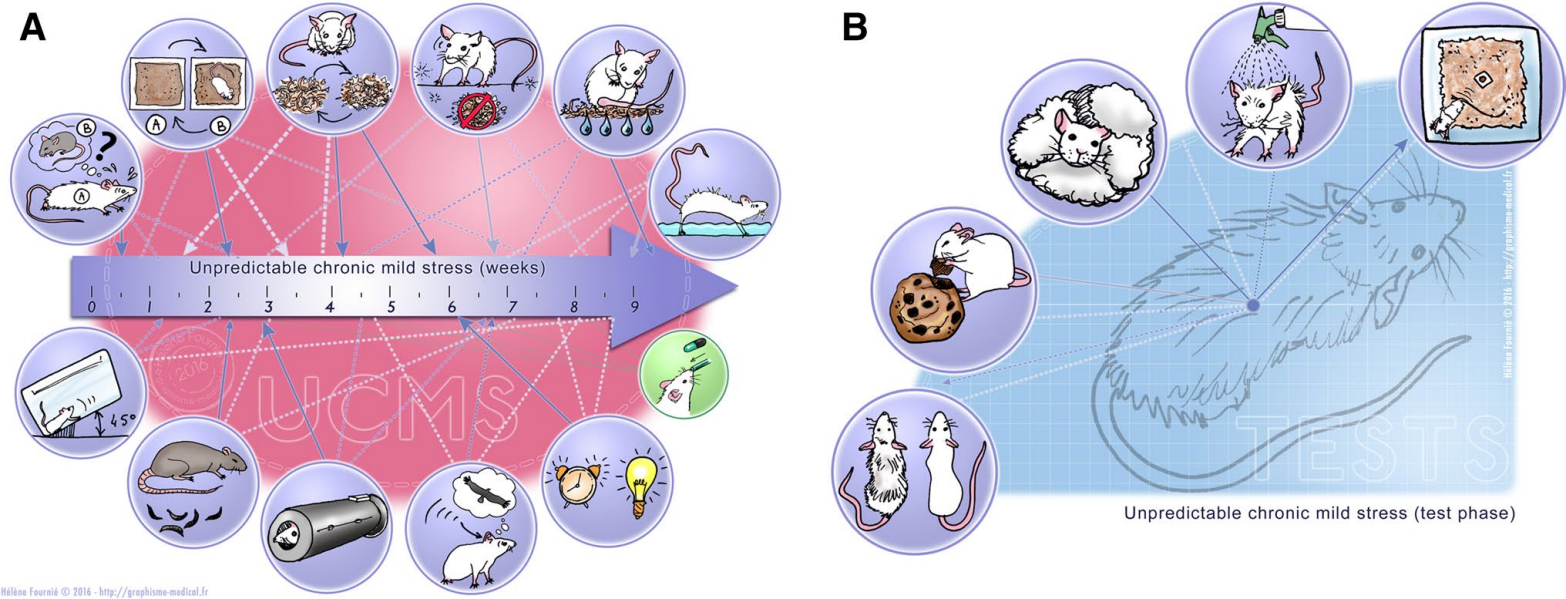
The unpredictable chronic mild stress (UCMS) model of depression. **a** Different stressors can be sued, including changes in lighting, contention in a small tube, introduction of rats faeces, cage tilting, social stress, cage changes, sawdust changes, no sawdust, humid sawdust, water in the cage. **b** Different endpoints can be measured after UCMS, including coat state, reward maze test, nest building, splash test, novelty suppression of feeding test

UCMS consists in subjecting rodents to a wide variety of socio-environmental stressors which have the following characteristics: (1) they are mild regarding their intensity (i.e. they never induce physical pain or food/water deprivation and none of the stressors can alone have durable repercussions on the mouse phenotype per se; (2) they are chronic, as they are repeated over weeks, while it is assumed that acute administration of such mild stressors would be ineffective; (3) they occur in an unpredictable way regarding the schedule (i.e. different over days/weeks, the duration of each individual stressor, the moment of the day when it is administrated, etc.). An example showing the type of stressors used is depicted in Fig. 3. After several weeks, this protocol induces deterioration in the coat state, decreased grooming in the splash test, anhedonia in the cookie test or in the sucrose preference test, for example. These changes are reversed after chronic ADs (for review, see: Nollet et al. 2013): usually, the ADs are administered once the first changes are observed, usually after 2 weeks of UCMS. This is done to mimic the clinical condition, in which treatments are administered solely once the symptoms have appeared, as its administration is supposed to induce recovery. This model induces a myriad of neurobiological effects that mirror changes seen in MDD, including a defect in the regulation of the hypothalamus–pituitary–adrenal (HPA) axis, decreased hippocampal neurogenesis, increased microglial activation, reduced 5-HT neurotransmission in the forebrain, reduced AC-cAMP-PKA signalling, mainly in frontal regions, a decrease in neurotrophins such as Brain-Derived Neurotrophic Factor (BDNF) in the hippocampus, decreased dendritic branching in the hippocampus and in some frontal regions, and also impaired LTP in the hippocampus–accumbens pathway (Segev et al. 2014) (for a review see Hill et al. 2012). In some cases, the reproducibility of this model has been discussed: an update on the validity and reliability of this model, as well as a comprehensive review of data on its underlying neurobiological basis and on its sensitivity to AD effects can be found in Willner (2017).

### Biological causation

In MDD, several neurobiological alterations have been observed (for reviews see Willner et al. 2013; Belzung et al. 2015), such as increased activity in cerebral networks (including the anterior cingulate cortex (ACC) and other parts of the PFC, the amygdala, the hippocampus, the nucleus accumbens and the habenula), neuroinflammation, dysregulation of the HPA axis causing hypercortisolemia, changes in the level of some neurotransmitters such as 5-HT, or polymorphism in some specific genes. However, it is still unclear whether these changes are correlates of MDD, or whether instead they cause MDD. Biological models of MDD are rooted in the rationale that these changes cause MDD and that therefore, by inducing these changes in animal models, it is possible to model the causes of MDD.

#### Lipopolysaccharide injection

The bacterial endotoxin, a single injection of lipopolysaccharide (LPS) usually from 0.5 to 0.83 mg/kg (O’Connor et al. 2009a; Ohgi et al. 2013; Walker et al. 2013b), is used to create an inflammation-related model of MDD that is expressed by behavioural changes, for example, decreased sucrose preference and increased despair behaviour. This is associated with increased brain expression of pro-inflammatory cytokines, such as IL-1β and TNF-α (Dantzer et al. 2008) and is reversed by ADs (Ohgi et al. 2013). Interestingly, the neurobiological changes extend beyond neuroinflammation, as decreased BDNF levels in the PFC and hippocampus together with increases in these levels in the NAc have been observed (Zhang et al. 2014), as well as increased corticosterone (Castanon et al. 2003) and changes in monoamines in corticolimbic structures (Sens et al. 2017).

#### Bacille Calmette–Guérin (BCG) administration model

Similar to LPS injection, a single injection of Bacille Calmette–Guérin (BCG) injected at a dose of 10^7^ or 10^8^ CFU (Moreau et al. 2008; O’Connor et al. 2009b), can induce chronic depressive-like behaviours associated with neuroinflammation. BCG is a pathogen inoculated as a vaccine to tuberculosis. Interestingly, chronic infection in animal models elicits a chronic inflammatory response which lasts until 1 month after administration in mice. This chronic inflammation induces changes in (1) appetite and body weight, (2) despair, reflected by increased immobility time in FST and TST (O’Connor et al. 2009b), (3) a decrease of motor activity and (4) anhedonia, measured with the sucrose preference test (Moreau et al. 2008). Additionally, chronic fluoxetine treatments reverse such depressive-like symptoms (Moreau et al. 2008; Saleh et al. 2014; Rana et al. 2016).

#### Bulbectomy

The olfactory bulbectomy procedure relies on bilateral surgical ablation of the olfactory bulbs in rodents. Two weeks later, rodents display hyperactive behaviours when placed in a novel environment as well as increased sensitivity to stress, disrupted sleep cycles and weight loss, transient anhedonia and despair, similar to that observed in MDD patients. At a neurobiological level, a progressive neural degeneration within regions of the corticolimbic networks to which the olfactory bulbs project has also been observed. Similarly, inflammation and increased corticosterone levels have been found (Yang et al. 2014) together with changes in serotoninergic neurotransmission (Riad et al. 2017). Chronic (2–4 weeks) but not sub-chronic or acute treatments with classical ADs counteract the behavioural changes (for a review, see Song and Leonard 2005).

#### Corticosterone administration

Corticosteroids are released after stress-induced HPA axis activation: under normal circumstances, this maintains homeostasis faced with adversity. However, under pathological conditions reflected by greater concentrations of glucocorticoids, brain damage can occur, such as decreased hippocampal neurogenesis, decreased dendritic branching in CA3 (McEwen 2000) and in the PFC (Liston et al. 2006; Popoli et al. 2011). Stress is a critical trigger for depression to develop (Checkley 1996) and MDD patients exhibit hypercortisolemia; therefore, current models consist in exposing animals to high levels of glucocorticoids in their drinking water or by injecting corticosterone to mimic chronic stress. Interestingly, chronic CORT treatment can elicit dysregulation of the HPA axis similar to that observed in MDD patients and behavioural changes such as increased immobility in the forced swim test, decreased grooming in the splash test, deterioration of coat state, anxiety-like behaviours in the NSF tests, open field and light/dark test, and anhedonia illustrated by decreased sucrose preference (Johnson et al. 2006; Gourley et al. 2008; David et al. 2009; Weng et al. 2016). In addition, animals exhibit a blunted hormonal response to stress (Johnson et al. 2006), increased activation of stress-sensitive brain regions, such as the PFC and hippocampus (Kinlein et al. 2015), neuroinflammation (Chabry et al. 2015), a decrease in hippocampal BDNF in some strains (Jacobsen and Mørk 2006) and changes in serotoninergic neurotransmission (Fairchild et al. 2003). It is worth to note, however, that the plasmatic levels of corticosterone observed after exogenous corticosterone administration do not exactly overlap with the corticosterone changes observed after chronic stress. Indeed, mice treated with exogenous corticosterone exhibit changes in the circadian rhythm of circulating corticosterone, with elevated corticosterone levels during the dark phase of the cycle and unchanged levels during the circadian nadir (Kinlein et al. 2015), as these nocturnal animals confine their drinking to the dark phase of the cycle.

#### Genetic models

Depression has a strong genetic component; many studies have, therefore, tried to modify the expression of genes associated with vulnerability to develop MDD. Many transgenic lines have been created targeting genes implicated in the serotoninergic and noradrenergic systems, and HPA axis regulation. Moreover, genetic models include lines selected according to their sensitivity to stress, such as Wistar Kyoto rats which show increased emotionality and reactivity to stress (Will et al. 2003; Nam et al. 2014) and high reactive mice which are selectively bred according to their sensitivity to restraint stress (i.e. contention) in terms of corticosterone releases (Touma et al. 2008). Indeed, mice exhibiting higher reactivity (HR) are hyperactive compared to intermediate (IR) and low reactive (LR) peers in some behavioural tests mimicking the pyschomotor agitation observed in some depressive patients (Touma et al. 2008). In addition, congenital learned helplessness rats (cLH), which fail to escape even without prior exposure to stress such as electric shocks (Vollmayr et al. 2004), show relevant neuronal changes similar to those found in major depression, with increased activity in the ACC and habenula, along with depressive-like behaviours (Winter et al. 2011; Li et al. 2011a). Another interesting approach is gene mutation using knockout mice (knocking out the 5-HT system: 5-HT transporter, 5-HT_1A_, 5-HT_1B_, 5-HT_2B_, P11; of the HPA axis: FKBP1 or CRHR1; other systems: CB1, OCT2, DBH, vGlut, or MIF). An extensive review of these different models is beyond the objective of this review, as they vary according to the model used and the system targeted. These animals all display some aspects of treatment resistance together with increased sensitivity to stress (for a review see Willner and Belzung 2015). It is to be mentioned, however, that depression is a multigenic disease, in which the effects of genes interact strongly with environmental factors. A recent impressive meta-analysis on 807,553 individuals (246,363 persons affected by MDD and 561,190 controls) identified 102 independent variants, 269 genes, and 15 gene-sets associated with MDD (Howard et al. 2019). Therefore, genetic models bearing single mutations on a given gene cannot recapitulate the genetic causation of MDD. Further on, such genetic manipulations should be associated with exposure to stressful environments to recapitulate the role of stress vulnerability in triggering the clinical conditions.

#### Optogenetic manipulation

Several optogenetic experiments have been effective in inducing depression-like behaviours, or in exacerbating susceptibility to stress. These approaches included inhibition of the somata of the anteroventral part of the bed nucleus of stria terminalis (Johnson et al. 2016), stimulation of pyramidal neurons from the ACC (Barthas et al. 2015), inhibition of medium spiny neurons from the NAc expressing D1 receptors (Francis et al. 2015) or chronic stimulation of the medial PFC (Ferenczi et al. 2016). Moreover, results have been provided by experiments targeting the VTA: chronic phasic stimulation of the VTA–NAc circuit during chronic social defeats (Wook Koo et al. 2016), inhibition of VTA (Tye et al. 2012), phasic stimulation of VTA in stress-susceptible mice, phasic stimulation of VTA–NAc neurons and inhibition of VTA–medial PFC neurons (Chaudhury et al. 2012) all induced stress-susceptible phenotypes, as well as acute enhancement of ventral hippocampus to the NAc (Zhang et al. 2015). On the other hand, other experiments enabled depressive-like behaviours to be alleviated or resilience in stressful situations to be elicited. This is the case after: activation of VTA (Tye et al. 2012), or of the projections from VTA to mPFC (Friedman et al. 2014), enhancing activity in D1-medium spiny neurons in the NAc (Francis et al. 2015), photostimulation of the projections from the ventromedial PFC to the dorsal raphe nucleus (Challis et al. 2014), attenuation of ventral hippocampus–NAc transmission (Zhang et al. 2015) or after chronically reactivating hippocampal cells associated with a positive memory (MacDonald et al. 2015). In general, these models recapitulate the dysfunction of a specific brain circuit associated with MDD, but not the whole range of MDD-related deficits. It is noteworthy that the effects of ADs have not been assessed in these optogenetic models.

## Validity of the classical models of MDD

Table 1 recapitulates the findings described above, answering the following questions: (1) does the model induce behavioural changes analogous to those seen in depressed individuals? Here, we focus on changes corresponding to core symptoms (anhedonia/sadness-related behaviours) as the other depressive-related behaviours are generally nonspecific. It is apparent that most models are able to elicit changes evoking a depressive-like symptomatology; (2) does it cause biological alterations identical to those seen in MDD patients? Again, one can see that in most cases, some biomarkers of MDD are present in the animal models. In some cases the biomarkers that have been tested encompass the alterations of several biological systems (for example the HPA axis together with neurotransmission and neurotrophins) and in others, only alterations in one system have been detected/assessed; (3) does the model respond to chronic AD? This has been assessed in most models.

**Table 1:** Current animal models of depression

Models	Core symptoms	Biological alterations	Antidepressant reversal
Induced by stress			
Early life adversity (Vetulani 2013; Willner and Belzung 2015)	Despair but not anhedonia, sleep disturbances, psychomotor abnormalities	Neurotrophin alteration, hypercorticolemia, inflammation, increased activity in the amygdala, altered noradrenergic neurotransmission	Response to chronic AD
Learned helplessness (Seligman 1972; Vollmayr and Henn 2003; Zazpe et al. 2007; Maier and Seligman 2016)	Anhedonia, despair, social withdrawal, eating behaviours abnormalities	Increased 5-HT neurotransmission and 5-HT1A receptor desensitisation, neurotrophin alterations, hypercortisolemia	Response to chronic AD
Social defeat (Golden et al. 2011)	Social withdrawal and anhedonia, body weight loss, eating behaviour abnormalities, psychomotor abnormalities	Inflammation, hypercortisolemia, corticolimbic alterations, neurotransmission alterations, neurotrophin alterations	Response to chronic AD and to ketamine
UCMS (Willner 2017)	Despair, anhedonia, apathy, sleep disturbance, psychomotor abnormalities, body weight loss	Inflammation, neurotrophins alterations, hypercortisolemia, corticolimbic alterations, neurotransmission alterations	Response to chronic AD and to ketamine
Neuroinflamation			
Lipopolysaccharide injection (O’Connor et al. 2009a; Kubera et al. 2013)	Anhedonia and despair	Inflammation, changes in neurotrophins, increased corticosterone, changes in neurotransmission	Response to chronic AD
Bulbectomy			
(Kelly et al. 1997; Song and Leonard 2005)	Transient anhedonia, despair	Changes in corticolimbic circuits, inflammation, increased corticosterone, changes in neurotransmission	Response to chronic but not acute AD
Drinking corticosterone			
(Gregus et al. 2005; Zhao et al. 2008)	Despair, sleep disturbance	Increased corticosterone, changes in corticolimbic brain areas, inflammation, changes in BDNF, changes in neurotransmission	Response to chronic AD and ketamine
Genetic models			
SERT-KO (Holmes et al. 2003; Lira et al. 2003)	Increased susceptibility, despair	Serotoninergic syndrome (reduced serotonergic cell number and firing rate)	Absence of effects of AD
5-HT receptor-KO			
5-HT1a (Pattij et al. 2002; Santarelli et al. 2003; Carnevali et al. 2012)	Increased anxiety and susceptibility to stress	Increased physiological responses to acute and chronic stress	No response to chronic SSRIs in 5HT1a KO mice but response in 5HT1b KO (Trillat et al. 1998; Mayorga et al. 2001)
5-HT1b (Vinkers et al. 2011)	Despair, anhedonia, increased susceptibility	Increased stress-induced autonomic and locomotor responses	
vGut1 (Garcia–Garcia et al. 2009; Venzala et al. 2012)	Higher sensitivity, despair	Changes in neurotransmission (glutamate, GABA)	Effects of acute and chronic AD are abolished
HPA axis			
FKBP1 KO (Gassen et al. 2014)	Higher sensitivity, despair	Changes in the autophagic pathway	Not tested
CRFR2 (Bale et al. 2000, 2002)	Despair	Increased stress-induced corticosterone levels	Decreased response to chronic AD
BDNf mutations (Chen et al. 2006; Chourbaji et al. 2011)	Anxiety-related behaviours	Affected serotoninergic and noradrenergic system	No response to chronic AD
Neurocircuit modifications			
BNST inhibition (Johnson et al. 2016)	Increased susceptibility, anhedonia, decreased motivation	Changes in neuronal activity changes in neurotransmission (dopamine, serotonin)	Not tested
Acc stimulation (Barthas et al. 2015)			
Inhibition of spiny cells in Nac (Francis et al. 2015)			
Chronic stimulation PFC (Ferenczi et al. 2016)			
Stimulation/inhibition VTA (Chaudhury et al. 2012; Tye et al. 2012; Wook Koo et al. 2016)			

This table summarises the most commonly used animal models of depression, focusing on the validity criteria: presence of the core behavioural symptoms of depression, neurobiological changes and response to antidepressant treatments

*AD* antidepressant drugs

Most current models satisfy all three validity criteria, as they induce some aspects of a depressive-like phenotype accompanied by neuronal changes that are reversed by chronic AD treatment. However, it is possible to highlight some exceptions concerning the models based on neurocircuit manipulations, which focus only on one precise area or a neuronal population altered in depression, but not on the disorder as a whole. Indeed, depression lies on a putative combination of factors and thus, there is no a unique, specific neuronal circuit involved in its pathophysiology, but rather a multiple alteration of intricate networks. This latter reason explains why current animal models based on neuronal manipulations of specific projections/brain areas can only unravel singular abnormalities of the disease, rather than providing a unified explanation of the pathological mechanisms at play in depression. Nevertheless, these kinds of models are useful to help understand the precise neuronal mechanisms involved in more restricted depression-like behaviours and physiological abnormalities, to define specific targets for treatments. The effects of chronic ADs have not been evaluated in these latter models.

Stress-induced models are probably the most commonly used. They have a strong construct validity given that it is well known that stress can trigger the development of this pathology and are, therefore, believed to recapitulate broader symptomatic dimensions and more comprehensive aspects of the depression-related neuropathology than most of the others animal models (Pittenger and Duman 2008). Besides, these models often provide two types of response, with resilient and susceptible animals, which could be of great interest to develop depression-related biological markers and resilience based on active coping mechanisms.

Furthermore, models based on biological causation rely on physiological alterations and genetic mutations observed in humans and involved in susceptibility to depression. These modifications include alterations in factors involved in the HPA axis and the neuroinflammation system, and also in neurotransmission. While treatments with corticosterone and LPS injection induce depression-like behaviours reversed by chronic ADs, genetic mutations alone hardly lead to depressive phenotypes; instead, they promote susceptibility, enabling the study of gene and environment interactions.

Another point to mention is that in most models, sex differences can be observed: this is of interest as in human MDD, the prevalence of the disorder is much higher in women. A complete picture of sex differences is beyond the scope of this manuscript, as the picture is rather complex. An extensive review of these findings can be found in Ma et al. (2019). Despite the development of multiple animal models, current research has failed to develop new treatment options for depression since the introduction of SSRIs in the late 1980s. A better understanding of the disorder and an accurate identification of biological markers are necessary. Indeed, more accurate animal models are required to improve research on MDD and its subtypes.

### Animal models of depression subtypes

Major depression is not a homogenous disorder, and it is diagnosed when patients display a certain number of symptoms included in a list. Consequently, two depressive patients can share just a few or only one symptom. Regarding this symptomatic heterogeneity, subtypes of depression have been proposed including melancholic depression and atypical depression.

Currently, melancholic depression is defined as the occurrence of a severe and pervasive anhedonia; psychomotor disturbance (agitation or reduction); vegetative disturbance with loss of weight, sleep disturbance (insomnia) and reduced libido; decreased mood reactivity to positive stimuli or conditions; higher cognitive impairment and often psychosis such as a feeling of guilt (Rush and Weissenburger 1994; Swartz et al. 2010; Uher et al. 2011; van Loo et al. 2012; Darcet et al. 2016). Although biological features are not taken into account for diagnosis, we can notice several abnormalities that seem more specific to melancholic depression: higher HPA axis reactivity and hypercortisolemia (Keck and Holsboer 2001; Touma et al. 2008; Swartz et al. 2010), disturbances in sleep architecture (Armitage 2007) and altered psychomotor activity assessed by the CORE scale (Parker and Hadzi-Pavlovic 1996). Regarding the criteria to meet melancholic depression, four putative animal models have been identified. The first involved mice selectively bred based on their corticosterone release to restraint stress (i.e. contention), hyper- and hypo-reactive mice were separated: mice exhibiting high corticosterone response were considered high reactive (HR) compared to mice showing intermediate or low corticosterone response, identified as intermediate and low reactive animals (IR and LR, respectively) (Touma et al. 2008). Compared to IR and LR, HR mice display lower body weight, disturbed sleep architecture, cognitive deficits, increased emotional reactivity, hyperactive coping-style and agitation. Such alterations recapitulate many characteristics of the melancholic subtype of depression. Interestingly, chronic antidepressant treatments result in an improvement of the ‘melancholic-like’ features of HR mice (Surget et al. 2016).The second model is based on the same idea: rats were bred for a trait related to emotionality in a stressful context, measured using locomotor activity in a novel environment to observe their emotional reactivity (Stedenfeld et al. 2011). Rats bred for their lower emotional reactivity (bLR) present much more severe anhedonia behaviours in a sucrose preference test after a UCMS protocol; this behaviour could be compared to the extreme anhedonia observed in melancholic depression. Moreover, it has been hypothesised that patients suffering from melancholic depression show reduced reward processing (Martin-Soelch 2009), which could explain severe anhedonia and psychomotor disturbance. Similarly, bLR rats show reduced dopaminergic transmissions in the NAc and decreased self-administration of cocaine (Davis et al. 2008). Other rat strains have been described as putative models of depression, like the Flinder Sensitive Line, which exhibits sleep disturbance, reduced appetite and psychomotor dysfunctions (Abildgaard et al. 2011; Overstreet and Wegener 2013), symptoms that are quite similar in melancholic patients. Moreover, after a UCMS protocol, these rats display anhedonia (Pucilowski et al. 1993), a key symptom of depression, and also a greater response to ADs in the FST (Overstreet and Wegener 2013), reflecting the potential relevance of this model for melancholic depression. Finally, a model has been developed which is based on abnormalities in GABAergic neurotransmissions involved in stress response and altered in depressed patients (Luscher et al. 2011). Thus, mice with deficits in γ-aminobutyric acid receptors show higher anxiety, depressive-like behaviours and HPA axis hyperactivity (Shen et al. 2010), similar to melancholic patients (Gold and Chrousos 1999). Moreover, this model meets criteria of predictive validity because chronic treatments with ADs, like desipramine can reverse depressive-like symptoms and normalise HPA axis activity (Shen et al. 2010). Taken together, these putative models could improve the comprehension of melancholic depression markers and help in the development of new targeted therapeutic strategies.

Atypical depression, another frequent subtype of depression, is diagnosed in approximatively 15–25% of the depressed population, whereas 20–30% exhibit melancholic symptoms (Gold and Chrousos 2002). Patients with atypical subtypes exhibit anhedonia and depressed mood. However, in contrast to melancholic subtypes, they show hypersomnia, a gain of weight, hyper-appetite, lethargy and higher reactivity to the environment (Gold and Chrousos 1999). Biological measurements in patients highlight a reduced HPA axis activity and CRH concentration, suggesting abnormal stress adaptation (Gold and Chrousos 1999, 2002). A relevant model for this subtype of depression should exhibit the same depressive phenotype and if possible the same biological abnormalities illustrated by reduced HPA axis reactivity. A putative model for atypical depression is the selected line of LR mice. As mentioned above, LR mice were selectively bred according to their reactivity to restrain stress; while the HR line has been proposed to model the melancholic subtype, the LR mice may meet some validity criteria for atypical depression. Indeed, LR mice show passive coping behaviours in a stressful environment, weight gain and reduced HPA axis activity (Touma et al. 2008), mimicking some symptoms associated with atypical depression. However, few animal models have been identified or developed for atypical depression. It could explain the lack of insight into biological markers of such depression subtypes. Further studies focusing on mechanistic aspects could improve our understanding of the pathophysiological and symptomatic characteristics underlying each subtype, to adapt patient treatment according to their specific diagnosis.

### Animal models of “Premenstrual dysphoric disorder”

Most described animal models are based on the use of males to avoid variability depending on the hormonal cycle. However, specific conditions actually rely on hormonal fluctuations, such as the premenstrual dysphoric disorder (PMDD) in females. Indeed, PMDD is a depressive disorder associated with the female premenstrual cycle. First classified in Annex B of the DSM IV—“criteria stets and axes provided for further study”—it was later included into the depressive disorder section of the DSM. PMDD is diagnosed when at least five symptoms from the following are present: mood swing, depression or sadness, anger or irritability, anxiety, anhedonia, difficulties in concentration, lethargy, abnormal appetite, changes in sleep, feeling overwhelmed or out of control and physical symptoms (tension, joint or muscle pain). These symptoms are recurring monthly after the ovulation phase and decrease in a few days after the onset of the menses. PMDD affects between 3 and 8% of women and causes severe daily-life functioning difficulties. Currently, most interventions are based on SSRIs and hormonal treatments, with the therapeutic distinction that SSRIs are not required on a daily basis but can be administered only during the luteal phase of the cycle. Because most of the animal models of depression are developed and studied with males, only few studies have explored PMDD and the precise mechanisms underlying the appearance of symptoms remain poorly understood. Nevertheless, some studies focus on the hormonal fluctuations and their consequences on behaviours of female rats, and found that approximatively 40% of the female Wistar rats exhibit higher aggressiveness when exposed to an unknown intruder during the non-receptive phase (metestrus and diestrus) compared to receptive phases (proestrus and estrus) (Ho et al. 2001; Schneider and Popik 2007a). The ovariectomy reduces such behaviours, which could be restored by the administration of steroid hormones such as estradiol and progesterone that are released during a functional estrus cycle. These hormonal fluctuations induce aggressive behaviours toward an intruder and could reflect irritability observed in women with PDDM. Moreover, females exhibiting aggressiveness also show depressive-like symptoms in the FST with increased immobility times (Schneider and Popik 2007a). Interestingly, the administration of fluoxetine reduces the aggressiveness, meeting the predictive validity criteria for this rodent model of “premenstrual irritability” in the resident–intruder test (Ho et al. 2001). Similarly, a fraction of cycling female Wistar rats (30%) exhibit enhanced burying behaviours during the metestrus phase, mimicking cycle-dependent irritability (Schneider and Popik 2007b, 2009). Additionally, a second rodent model has been developed, based on progesterone fluctuations: indeed, progesterone withdrawal is a model in which long-term administration of exogenous steroids is abruptly stopped (Li et al. 2012, 2013; Islas-Preciado et al. 2016). Progesterone withdrawal in female rats can elicit robust and reproducible depression-like behaviours such as increased despair and anhedonia, reversed by tricyclic antidepressant amitriptyline and vortioxetine (Li et al. 2012, 2013).

### Animal models of treatment-resistant depression and recurrent depression

#### Treatment-resistant depression

The current standard care for MDD is pharmacological treatment with the administration of ADs such as selective serotonin reuptake inhibitors (SSRIs), selective norepinephrine or dopamine reuptake inhibitors. However, these treatments have some limitations as they do not induce complete remission in all patients, with only one-third of patients achieving remission after a treatment with a standard SSRI (Trivedi et al. 2006). Moreover, some patients are resistant to these compounds. This inadequate response to pharmacological treatments, referred to as treatment-resistant depression (TRD), is diagnosed when there is a failure to respond to two or more courses of ADs (Souery et al. 2006).

While current animal models are good tools to understand the underlying mechanisms of conventional ADs, the discovery of new targets would benefit patients suffering from TRD. To that purpose, treatments acting on different mechanisms than those involved in current AD drugs are needed. However, as animal models of MDD are also based on predictive validity, that is to say on response to conventional ADs, they cannot be used as models of TRD, and alternative models are required. For example, as heightened vulnerability to depression is associated with resistance to AD treatments (Willner et al. 2013, 2014), relevant models for TRD may be based on increased vulnerability rather than on stress-induced depression (Willner and Belzung 2015). Moreover, a relevant model of TRD should be resistant to current ADs but not to non-conventional treatments like DBS, TMS or ketamine, as these treatments have been demonstrated to display higher effectiveness in human patients suffering from TRD than conventional ADs (Höflich et al. 1993; George et al. 2000; Mayberg et al. 2005; aan het Rot et al. 2010; Janicak et al. 2010; Murrough et al. 2013). Based on these criteria, several animal models have been proposed to study resistance to treatments in depression, including separation into responders and non-responders to ADs, administration of treatments that render the animals resistant, and identification of genetic models that show antidepressant resistance.

Indeed, in UCMS, one of the most commonly used depression models, different responses to antidepressant treatments have been observed, with some rodents undergoing antidepressant-induced recovery, while others being refractory to the treatment. One study demonstrated that after chronic stress procedures, approximatively 30% of rodents were resilient, and in the other 70% of susceptible rodents, only half were sensitive to drugs, while the other half were resistant (Bisgaard et al. 2007; Javanbakht et al. 2011; Christensen et al. 2011). In another study, animals that were on a high-fat diet during multiple UCMS procedures became resistant to a SSRI (fluoxetine) treatment (Isingrini et al. 2010). Although this model does not validate the criteria of vulnerability to depression without stress exposure, it gives us a relevant tool to analyse the mechanism underlying TRD when compared to depressive-like rodents sensitive to ADs. Furthermore, studies have shown similarities in gene expression between resilient and depressive-like rodents which respond to AD and between non-responders and anhedonic rodents, suggesting that antidepressant mechanisms of action emulate endogenous stress-coping strategies (Christensen et al. 2011).

Social defeat and chronic corticosterone administration models have also been used in the same manner to separate responder and non-responder individuals. Here, the brain reward threshold (the current intensity inducing a response), using the intracranial self-stimulation procedure, is elevated after social defeat, and ADs decrease this threshold in only 50% of rodents (Der-Avakian et al. 2014). The same pattern was observed in a chronic corticosterone model, which increases latency in the novelty-suppressed feeding test, induces anhedonia and increases immobility in the forced swim test (Murray et al. 2008; David et al. 2009). However, in response to fluoxetine, a bimodal distribution was observed with responders and non-responders (David et al. 2009). Furthermore, as mentioned above, a relevant TRD model should respond to non-classical antidepressant treatments. Indeed, treatment with a glutamatergic regulator such as ketamine and a modulator of AMPA receptors, induced antidepressant-like effects in rodents after chronic stress (Li et al. 2011b), chronic corticosterone administration (Mendez-David et al. 2017) and social defeats (Donahue et al. 2014; Yang et al. 2016; Dong et al. 2016). Similarly, deep brain stimulation showed efficacy (Dandekar et al. 2018) in a chronic stress model (Hamani et al. 2012; Bambico et al. 2015), more specifically in treatment-resistant mice (Dournes et al. 2013), as well as in a social defeat model (Veerakumar et al. 2014).

Other models have tried to induce resistance with treatments of interleukine 6 (Sukoff Rizzo et al. 2012) or chronic adrenocorticotropic hormone (ACTH) (Kitamura et al. 2002; Walker et al. 2013a). Associating chronic mild stress and an injection of lipopolysaccharide (Wang et al. 2011) or a high fat diet (Isingrini et al. 2010) can also be relevant. Furthermore, models using genetically modified animals or those selected for heightened vulnerability could be fit for purpose. Among many examples, mutation of genes implicated in depression induce depression-like phenotypes and resistance to ADs: serotonin transporter 5HTT knockout mice exhibit depression-like behaviours in FST which can or cannot be reversed by fluoxetine (Holmes et al. 2003), serotonin receptor 5HT1a KO mice do not respond to SSRIs (Santarelli et al. 2003), p11 knockout mice show reduced sensitivity to fluoxetine in NSF (Egeland et al. 2010) and finally mutation of adrenergic receptor alpha2A (Schramm et al. 2001) and dopamine beta-hydroxylase knockout mice (Cryan et al. 2001, 2004) are less sensitive to conventional drugs in FST. Moreover, many selected lines show heightened vulnerability to stress, although at an insufficient level to meet the criteria for a TRD model, such as the HAB rats (insensitive to three ADs) and Flinders Sensitive rats, which are resistant to escitalopram in FST after depression-like states induced by maternal separation (for a review see Caldarone et al. 2015). However, each of these models, particularly those with targeted mutations, might only be a relevant model for a specific cause of TRD: for example, the resistance shown by the 5HTT mutant might be an adequate model for resistance related to a polymorphism of the 5HTT, but not for other forms of TRD.

#### Recurrent depression

A remarkable characteristic of MDD is its recurrence, with almost half of depressive patients having at least two episodes of depression and with a gradually increasing susceptibility to recurrence after successive episodes. Indeed, after a second episode, approximatively 80% of the patients will develop new episodes (Maj et al. 1992; Post 1992; Mueller et al. 1999). Recurrent depression is defined as the occurrence of another episode after recovery and it must be differentiated from relapse, which is the appearance of a novel episode during remission and before recovery (Frank et al. 1991).

Few animal models specific to recurrent depression have been proposed. Some studies have shown that an initial experience of chronic stress in rats induce susceptibility and faster onset of depressive-like behaviours when re-exposed to stress (Remus et al. 2013; Alves et al. 2017). Moreover, in this model, rats are re-exposed to stress to trigger another depressive episode after a recovery phase, during which the increase of sucrose consumption backs down to baseline, meeting the criteria for recurrent depression.

Interestingly, when associated with a high-fat diet, two consecutive episodes of UCMS induced a phenotype that was resistant to antidepressants, as in human recurrent depression (Isingrini et al. 2010). However, these models are not optimal as in the human condition, recurrent episodes become independent of a stress-related aetiology (Willner and Belzung 2015), while rodent models do not present recurrence of depressive-like symptoms unless they are submitted to new stress.

## Modelling high risk of depression: resilience and susceptibility

Modelling high risk of depression could enable the effectiveness of prevention strategies to be assessed. In fact, after a stressful event most of the population does not develop a pathological state such as post-traumatic stress disorder or depression, and this is related to resilience. Some factors like cognitive flexibility (Yehuda et al. 2006) might play a protective role. Moreover, research on genetic and environmental interactions suggests that positive social support could promote resilience in children with higher vulnerability induced by abuse during infancy (Kaufman et al. 2004, 2006), and in the same way, rodent models have shown that enrichment of the environment can prevent the appearance of depressive-like episodes (van Praag et al. 2000; Mohammed et al. 2002; Francis et al. 2002; Hattori et al. 2007; Laviola et al. 2008; Schloesser et al. 2010; Hendriksen et al. 2012; Branchi et al. 2013). However, biological features underlying resilience are poorly known. Thus, instead of treating the consequences of the pathological state, signs, and symptoms, a better understanding of the aetiology could be more relevant to treat the causes. To that purpose, new animal models need to be developed to study biological biomarkers involved in vulnerability and resilience to depression to help counter the potential occurrence of depressive episodes and further promote its prevention. One possibility in current animal models is to segregate individuals which do not develop a depression-like phenotype following stress: in UCMS (Strekalova et al. 2004; Bergström et al. 2007, 2008), social defeat (Krishnan et al. 2007; Tse et al. 2014) or LH. Indeed, variability in sensitivity to the UCMS protocol can be observed between strains (Castro et al. 2012; Ducottet et al. 2004; Ducottet and Belzung 2005), or in relation to emotionality in rats (Stedenfeld et al. 2011), and submissiveness in mice (Strekalova et al. 2004). Furthermore, UCMS induces depressive-like behaviours in only 70% of rodents, with the remaining approximatively 30% of the population considered as resilient (Bergström et al. 2008; Delgado y Palacios et al. 2011; Russo et al. 2012). These resilient mice showed no decrease in hippocampal volume or alteration in glutamate metabolism after chronic stress (Delgado y Palacios et al. 2011). Moreover, activity in the amygdala, habenula or infralimbic was increased selectively in susceptible mice (Febbraro et al. 2017) and this could lead to identify potential biomarkers. Similarly, research showed that almost half of the experimental animals did not respond to chronic social defeat and thus were resilient to stress: these mice showed an increase in hippocampal volume and a higher NMDA receptor function in this region compared to susceptible mice (Tse et al. 2014, 2019). It also appears that some changes in gene expression and chromatin structure were present only in resilient mice (Krishnan et al. 2007; Wilkinson et al. 2009; Mallei et al. 2018). Until recently, most studies on resilience focused on the absence of biomarkers of depression rather than on active mechanisms promoting resilience. However, the characterization of these protective factors would be beneficial to enhance knowledge of the neuronal basis of the disorder and how to treat it. Thus, a comparison between resilient and susceptible animals in classical models could further determine biomarkers of resilience and susceptibility to depression, which could then be measured and targeted to improve current treatments and facilitate prevention when possible.

## Future challenges

Recently, new approaches to psychiatric pathologies have come to light. Historically and since the introduction of the DSM III, psychiatric disorders have been classified according to the disease categories (depression, schizophrenia, etc.) and animal models rely on this approach. However, one of the issues of the current classification is the high prevalence of comorbidities, and hence the difficulty to maintain strict boundaries between disorders. Furthermore, a debate remains regarding the validity of this approach because it does not reflect the complexity and intensity of symptoms in patients. Around 10 years ago, the National Institute of Mental Health of the United States launched the Research domain criteria (RDoC), a framework to classify patients according to specific dimensions, including behavioural/cognitive aspects as well as impaired brain systems (Casey et al. 2013). This approach seeks to identify the altered dimensions and focuses on the identification of neuronal correlates. Five dimensions corresponding to the main cognitive/affective systems which are altered have been proposed: negative valence system; positive valence system; cognitive system; arousal/regulatory system and finally social process system. Each dimension also corresponds to a neural system (Insel et al. 2010). Indeed, the RDoC assumes the idea that psychiatric disorders are based on neurobiological alterations that can be measured. Although the RDoC approach is still nascent, this framework will encourage researchers to identify new biological markers that could help the development of new treatments. The use and development of animal models enabling the corresponding RDoC to be assessed will be one of the main challenges in the future. The definition and use of clear biological measures could lead to a more accurate definition and characterization of depressive disorders and animal models could be one of the keys to understanding the neuro-mechanisms of the disease. However, this approach has also its pitfalls as RDoC frame assumes the idea that the symptoms observed in a transnosographic way do really overlap, which is not the case. For example, the anhedonia displayed by depressive patients does not overlap with the anhedonia exhibited by schizophrenic patients (Culig and Belzung 2016).

Another important point to mention here is that in some case, the animal models are not the sole to blame for the lack of innovation in AD research. Indeed, in some case, failure is also related to a poor design of the clinical trials, which do not appropriately back-translate the findings from the animal models (see Belzung 2014) or to the lack of innovation of the Food and Drug Administration guidelines for approving new antidepressants (guidelines have remained unchanged for the past 30 years) (Hanrahan and New 2014).

## Conclusion

In conclusion, in this review, we have shown that the majority of the animal models of depression respect validity criteria, as defined by construct, face and predictive validity. Indeed, they mimic depressive phenotypes and induce neuronal changes similar to those observed in humans, reversed by classical ADs. However, to date, this approach has failed to lead to the development of new treatments and the biological mechanisms of depression are still poorly understood. Thus, future research should focus not only on the presence or absence of depressive symptoms but also on the biological features underlying the clinical signs. More importantly, animal models should respect human heterogeneity, to improve treatments by defining accurate targets.
